# Prediction of tumor progression in intermediate and advanced hepatocellular carcinoma undergoing TACE combined with targeted immunotherapy

**DOI:** 10.3389/fonc.2026.1678771

**Published:** 2026-04-24

**Authors:** Lina Sun, Yanqiu Li, Qiang Zhao, Ke Shi, Xue Yang, Qun Zhang, Guiqin Zhou, Ying Feng, Xianbo Wang, Yao Liu

**Affiliations:** 1 Center of Integrative Medicine, Beijing Ditan Hospital, Capital Medical University, Beijing, China; 2 Department of Digestive Diseases, The First Affiliated Hospital of Henan University of Chinese Medicine, Zhengzhou, China

**Keywords:** hepatocellular carcinoma, immune checkpoint inhibitors, immunotherapy, nomogram, targeted therapy, transcatheter arterial chemoembolization

## Abstract

**Background:**

Transcatheter arterial chemoembolization (TACE) combined with targeted immunotherapy have become the standard first-line treatment strategy for intermediate and advanced hepatocellular carcinoma (HCC), but some patients still cannot benefit from this treatment. This study aimed to construct a prediction model based on a cohort of HCC patients to guide individualized treatment decisions.

**Methods:**

A total of 243 intermediate and advanced HCC patients who received TACE combined with targeted immunotherapy from January 1, 2019 to March 31, 2024 were retrospectively enrolled. The optimal prognostic factors were screened by Cox regression analysis and least absolute shrinkage and selection operator (LASSO) regression. A nomogram model for predicting the probability of radiologic progression-free survival at 6-, 12-, and 24-month was constructed based on the screened risk factors, and the model performance was evaluated by calibration curve, decision curve analysis, and restricted cubic spline analysis.

**Results:**

Multivariate COX analysis showed that total bilirubin, D-dimer (DD) and portal vein tumor thrombosis (PVTT) were independent risk factors affecting the tumor progression of patients. LASSO regression screened out 10 key prognostic factors: aspartate aminotransferase, total bilirubin, albumin (ALB), white blood cell count, DD, alpha-fetoprotein, CD4+ T cell count, PVTT, tumor number (≥3) and lymph node invasion. Compared with the Cox regression model, significant advantages in screening prognostic factors were demonstrated by the LASSO regression model. Therefore, the risk factors identified by LASSO regression were chosen to construct a nomogram prediction model for subsequent analysis. The model showed good discrimination ability (Log rank P<0.05), calibration ability and net benefit of clinical strategy in both the training and validation group. Restricted cubic spline analysis found that ALB level and DD were significantly nonlinearly correlated with patient prognosis.

**Conclusion:**

This study constructed a multidimensional prediction model for tumor progression in patients with intermediate and advanced HCC. It was helpful to screen the best benefit population and optimize the treatment strategy.

## 1 Introduction

Hepatocellular carcinoma (HCC) is the most common primary liver malignancy worldwide and one of the leading causes of cancer-related death (1). The incidence and mortality of HCC in Asia are among the highest in the world, accounting for approximately 72% of the total cases worldwide (2), and most patients are already in the advanced stage when diagnosed (3). According to the Barcelona Clinic Liver Cancer (BCLC) staging system, Stage B (intermediate-stage) HCC refers to multinodular tumors without vascular invasion or extrahepatic spread in patients with well-preserved liver function (Child-Pugh A-B grade), while BCLC Stage C HCC refers to patients with vascular invasion or distant metastasis but still maintain good liver function (4–6). Such patients have a poor prognosis, with a historical median survival of only 3–6 months (7).

The treatment landscape for BCLC Stage B and C HCC has undergone significant evolution over the past decade. Traditionally, transarterial chemoembolization (TACE) has been established as the standard treatment for patients with BCLC Stage B HCC. BCLC Stage B represents a heterogeneous population with varying tumor burden, liver function, and response to treatment. Patients with BCLC Stage B HCC who are unsuitable for or refractory to locoregional therapies should be offered systemic therapy. For patients with BCLC Stage C HCC, sorafenib, as the only approved first-line systemic treatment option from 2007 to 2017, brought an overall survival (OS) benefit to patients with advanced HCC (8, 9). Another potent tyrosine kinase inhibitor, lenvatinib, was launched in 2018 and has played a leading role in improving clinical outcomes in advanced HCC (10).

In recent years, with the emergence of targeted therapy and immune checkpoint inhibitors (ICIs), the treatment landscape of advanced liver cancer has undergone some changes (11, 12). These drugs are superior to sorafenib in terms of OS and progression-free survival (PFS) in patients with advanced HCC (13). For patients with BCLC-C HCC, the combination of targeted drugs and ICIs is recommended as the first-line standard treatment (14). Currently, the targeted drugs approved for advanced HCC in China mainly include lenvatinib, sorafenib, donafenib, etc.; ICIs include sintilimab, camrelizumab, toripalimab, etc. Among them, lenvatinib combined with sintilimab, as a combination with a high domestic production rate, has been widely used in China for the first-line treatment of advanced HCC.

In patients with unresectable HCC, lenvatinib combined with pembrolizumab can achieve an objective response rate (ORR) of 34.1% (15), and maintain health-related quality of life during the first-line treatment (16). And the combination of lenvatinib and sintilimab can prolong the ORR to 36.1%, and the disease control rate is as high as 94.4% (17, 18). Although targeted immune combination therapy has significantly improved the survival of patients, some patients still cannot benefit from the treatment, and the drug cost is high and the incidence of adverse reactions cannot be ignored (19). Therefore, developing an effective prediction model to screen the best benefit population has become a hot topic in current research and a problem that needs to be solved urgently in clinical practice.

At present, there are many models for predicting the effect of immunotherapy in patients with HCC, including prediction models based on peripheral blood biomarkers, tumor microenvironment characteristics, and imaging characteristics (20–23). However, most of these models are based on single-dimensional indicators, have limited predictive value (24), and are mainly targeted at single immunotherapy, lacking specific evaluation for patients undergoing the combined modality of TACE with targeted immunotherapy, which represents a distinct therapeutic context. By integrating baseline clinical characteristics and imaging features, we aim to develop a clinically applicable tool that can identify optimal candidates for TACE combined with lenvatinib and sintilimab, thereby facilitating individualized treatment decisions and improving resource allocation.

## 2 Methods

### 2.1 Study subjects

This study retrospectively enrolled patients with intermediate and advanced HCC undergoing TACE combined with targeted immunotherapy at Beijing Ditan Hospital from January 1, 2019 to March 31, 2024. The inclusion criteria were as follows: (I) aged between 18 and 80 years; (II) diagnosis of HCC confirmed by clinical criteria in accordance with AASLD guidelines (14), with the following stages: BCLC stage C (extrahepatic metastasis or macrovascular invasion), or BCLC stage B (multiple nodular type) not suitable for or ineffective for TACE monotherapy or refractory to prior TACE treatment; (III) received TACE combined with lenvatinib and sintilimab; (IV) availability of complete clinical and follow-up data. Exclusion criteria were: (I) patients with other non-HCC primary liver malignancies; (II) prior exposure to ICIs or multi-targeted tyrosine kinase inhibitors (TKIs) before the current treatment regimen; (III) evidence of uncontrolled or significant cardiovascular diseases, including recent myocardial infarction (within 6 months), unstable angina, congestive heart failure (New York Heart Association class III or IV), or significant arrhythmias; (IV) active gastrointestinal bleeding or untreated high-risk esophageal or gastric varices as assessed by upper endoscopy within 6 months prior to treatment; (V) known history of autoimmune disease requiring systemic immunosuppressive therapy; (VI) active infection including hepatitis B virus (HBV) with high viral load (HBV DNA > 2000 IU/mL without antiviral treatment), hepatitis C virus (HCV)with detectable HCV RNA, or human immunodeficiency virus infection; (VII) lost to follow-up. This study was conducted in accordance with the ethical principles outlined in the Declaration of Helsinki. This study was approved by the Ethics Committee of Beijing Ditan Hospital.

### 2.2 Treatment plan

Eligible patients received a combination regimen of TACE plus lenvatinib and sintilimab. Before TACE, superior mesenteric artery angiography and common hepatic artery angiography were performed to assess tumor blood supply, vascular anatomy, and tumor extent. After local anesthesia, a 5F catheter was inserted into the abdominal aorta via the superficial femoral artery using the Seldinger technique. During hepatic arteriography, catheters were inserted into the celiac and superior mesenteric arteries under fluoroscopy. The feeding arteries were then identified, and the tumor and surrounding vascular anatomy were stained. A microcatheter was then inserted into the feeding artery via the catheter. A combination of superfluid iodized oil (5–10 ml), lobaplatin (20–40 mg), and pirarubicin (10–30 mg) was then infused into the tumor. If a significant arterioportal (AP) shunt was present, embolization with gelatin sponge particles was performed to occlude the shunt. Additional angiography was performed before the procedure to ensure complete occlusion of the feeding artery. TACE was repeated on an on-demand basis, typically every 6–8 weeks, guided by follow-up imaging assessments (contrast-enhanced CT or MRI) and tumor marker dynamics, provided that the patient’s liver function and performance status were deemed adequate for repeat intervention. Lenvatinib was taken orally, 12 mg/day for patients weighing ≥60 kg and 8 mg/day for patients weighing <60 kg. Sintilimab was injected intravenously at a dose of 200 mg once every 3 weeks. Treatment-related adverse events were monitored throughout the study period. Dose adjustments or temporary interruptions of lenvatinib were permitted based on the severity of adverse events, following standard clinical practice guidelines. Sintilimab administration could be delayed or discontinued in the event of immune-related adverse events (irAEs) requiring prolonged immunosuppression or if irAEs did not resolve to Grade 1 or lower within a specified timeframe. Treatment was continued until any of the following criteria were met: (I) radiological or clinical disease progression as per RECIST 1.1; (II) the occurrence of unacceptable toxicity (defined as Grade 3 or 4 adverse events that did not improve to Grade ≤1 after dose interruption or optimal supportive care); (III) withdrawal of consent by the patient; or (IV) decision by the treating physician based on a significant decline in performance status or other clinical considerations that precluded continuation of therapy.

### 2.3 Follow-up and efficacy evaluation

Patients underwent imaging assessment (enhanced CT or MRI) every 6–8 weeks after the start of treatment, and the efficacy was evaluated according to the Response Evaluation Criteria in Solid Tumors (RECIST) version 1.1. The primary endpoint for this prognostic analysis was time to radiologic disease progression, defined as the interval from treatment initiation to the first documentation of progressive disease per RECIST 1.1, including the appearance of new lesions or unequivocal progression of existing target or non-target lesions. OS is defined as the time from the start of treatment to death due to any cause or the last follow-up. The follow-up deadline was March 31, 2024.

### 2.4 Data collection

Baseline clinical data of patients were collected, including: (I) demographic characteristics: age, gender; (II) liver function indicators: total bilirubin (TBIL), albumin (ALB), aspartate aminotransferase (AST), alanine aminotransferase (ALT), γ-glutamyl transpeptidase (GGT); (III) blood cell count: white blood cell count (WBC), neutrophil count, lymphocyte count, platelet count, and NLR; (IV) coagulation function: prothrombin time (PT), prothrombin activity (PTA), international normalized ratio (INR), D-dimer (DD); (V) tumor characteristics: AFP level, lymph node invasion, portal vein tumor thrombus (PVTT), hepatic vein tumor thrombus (HVTT), inferior vena cava tumor thrombus (ICVTT), tumor number, maximum tumor diameter, and presence of cirrhosis; (VI) immune function: CD4+ T cell count, CD8+ T cell count, and lymphocyte count.

### 2.5 Statistical analysis

Measurement data were expressed as mean ± standard deviation or median (interquartile range) according to distribution characteristics, and t-test or Mann-Whitney U test was used for inter-group comparison; count data were expressed as frequency (percentage) and compared by χ² test or Fisher’s exact test. Patients were randomly assigned to the training group and the validation group in a ratio of 8:2.

Univariate and multivariate Cox proportional hazard regression models were used to analyze the independent risk factors, and the results were expressed as hazard ratio (HR) and its 95% confidence interval (CI). To avoid multicollinearity problems and screen the optimal prognostic factors, we used the least absolute shrinkage and selection operator (LASSO) regression analysis. The optimal λ value was selected by 10-fold cross validation to determine the variables entering the prognostic model.

Based on the clinical risk factors screened by LASSO regression, a nomogram model was constructed to predict the 6-, 12-, and 24-month survival probability of HCC patients. According to the total points calculated by the nomogram, the patients were divided into low-risk group and high-risk group, and the survival curve was drawn by Kaplan-Meier method to compare the survival difference between the two groups (Log-rank test).

The calibration curve was used to evaluate the accuracy of the model in predicting the 6-, 12- and 24-month survival probability in the training cohort and the validation cohort. The clinical practical value of the model was evaluated by decision curve analysis (DCA), and the net benefit (Net Benefit) under different threshold probabilities was calculated. Restricted Cubic Spline (RCS) analysis was applied to evaluate the nonlinear relationship between clinical biochemical indicators and patient prognosis. The relationship pattern between each parameter and prognosis was determined by calculating the overall *P* value (*P* overall) and nonlinear *P* value (*P* nonlinear).

All statistical analyses were performed using R software (version 4.3.1), and *P* < 0.05 was considered statistically significant.

## 3 Results

### 3.1 Baseline clinical characteristics

**Figure 1:**
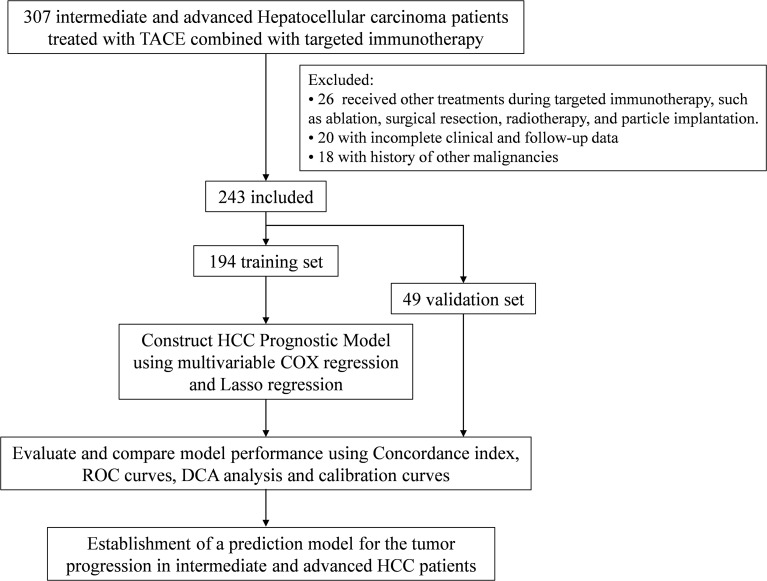
Patient selection flowchart. Flowchart showing the selection process of 243 intermediate and advanced HCC patients undergoing TACE combined with targeted immunotherapy.

A total of 243 intermediate and advanced HCC patients undergoing TACE combined with targeted immunotherapy were included in this study (**Figure 1**), including 194 in the training cohort and 49 in the validation cohort. During follow-up, 106 progression events were observed in the training cohort. In the overall cohort, 23.9% were female, the median age was 61 (54-68) years, and the follow-up time was 196 (105-359) days. Liver function, blood cell counts, and coagulation function are shown in **Table 1**. In terms of tumor characteristics, AFP was 132.39 (6.63-2000) μg/L, 40.3% of patients had PVTT, 14.0% had HVTT, 8.6% had ICVTT, 46.1% of patients had ≥3 tumors, 23.0% of patients had a maximum tumor diameter of ≥ 5 cm, 24.3% of patients had lymph node invasion, and 81.9% of patients had cirrhosis. In terms of immune function, the CD8+ T cell count was 332.6 (327.6-332.6), and the CD4+ T cell count was 510 (439-510). There were no statistical differences between the training cohort and the validation cohort in most baseline characteristics (*P* > 0.05), but there were significant differences in lymphocyte count (*P* = 0.008), NLR (*P* = 0.020), PT (*P* = 0.014), PTA (*P* = 0.026), and INR (*P* = 0.019).

**Table 1:** Baseline clinical characteristics of HCC patients.

Variables	Total cohort (n=243)	Training cohort (n=194)	Validation cohort (n=49)	*P* value
Sex (% female)	58 (23.9)	46 (23.7)	12 (24.5)	0.909
Age, years	61 (54, 68)	60 (54-69)	61 (55-68)	0.655
Follow-up time, days	196 (105-359)	208.5 (116.75-359)	171 (83-342)	0.431
AST, U/L	32.4 (23.1-51.75)	31.8 (22-50.6)	32.5 (24.1-55)	0.799
ALT, U/L	35.2 (24.65-56.1)	35.2 (24.4-58.7)	35.4 (24.8-52.7)	0.754
TBIL, μmol×L^-1^	16.5 (11.2-23)	16.5 (11-23)	17.4 (12.5-23.1)	0.702
ALB, g×L^-1^	37.45 ± 5.06	37.69 ± 4.97	36.48 ± 5.32	0.706
GGT, U/L	113.3 (46.9-113.3)	113.3 (46.4-113.3)	103 (47.7-113.3)	0.719
ALP, U/L	134.3 (86.8-134.3)	143.3 (89.1-134.3)	119.3 (83.6-134.3)	0.324
CRP	29.9 (5.35-29.9)	29.9 (5.4-29.9)	29.9 (5.3-29.9)	0.436
WBC, 10^9^×L^-1^	4.75 (3.65-6.64)	4.69 (3.6-6.55)	5.38 (3.75-6.73)	0.494
NE, 10^9^×L^-1^	3.01 (2.23-4.25)	3.01 (2.24-4.23)	2.94 (1.93-4.48)	0.923
LE, 10^9^×L^-1^	1.06 (0.75-1.47)	1.0 (0.72-1.41)	1.32 (0.89-1.71)	0.008
NLR	2.84 (2.0-4.28)	2.9 (2.09-4.43)	2.52 (1.59-3.7)	0.020
PLT, 10^9^×L^-1^	135 (89.5-192)	133.5 (91-189)	148 (79-225)	0.433
PT, s	12.7 (11.65-13.9)	12.5 (11.5-13.6)	13.3 (12.2-14.2)	0.014
PTA, %	80 (71-90)	81 (72-92)	76 (68-86)	0.026
INR	1.17 (1.07-1.28)	1.16 (1.06-1.26)	1.22 (1.13-1.31)	0.019
DD	1.27 (0.58-1.92)	1.33 (0.57-1.92)	1.0 (0.68-1.76)	0.263
AFP, μg/L	132.39 (6.63-2000)	128.21 (6.76-2000)	159.71 (5.29-2000)	0.988
CD8+ T cell	332.6 (327.6-332.6)	332.6 (326.6-332.6)	332.6 (329.6-332.6)	0.603
CD4+ T cell	510 (439-510)	510 (432-510)	510 (451-510)	0.562
CR	67 (56.1-75.4)	67.1 (56-76.5)	67 (59.1-73.1)	0.579
PVTT				0.566
NO	145 (59.7)	114 (58.8)	31 (63.3)	
YES	98 (40.3)	80 (41.2)	18 (36.7)	
HVTT				0.392
NO	209 (86)	165 (85.1)	44 (89.8)	
YES	34 (14)	29 (14.9)	5 (10.2)	
ICVTT				0.663
NO	222 (91.4)	178 (91.8)	44 (89.9)	
YES	21 (8.6)	16 (8.2)	5 (10.2)	
Tumor number				0.073
< 3	131 (53.9)	99 (51)	32 (65.3)	
≥ 3	112 (46.1)	95 (49)	17 (34.7)	
Tumor diameter, cm				0.788
< 5	187 (77)	150 (77.3)	37 (75.5)	
≥ 5	56 (23)	44 (22.7)	12 (24.5)	
Lymph node invasion				0.738
NO	184 (75.7)	146 (75.3)	38 (77.6)	
YES	59 (24.3)	48 (24.7)	11 (22.4)	
Liver cirrhosis				0.640
NO	44 (18.1)	34 (17.5)	10 (20.4)	
YES	199 (81.9)	160 (82.5)	39 (79.6)	

ALT, alanine aminotransferase; AST, aspartate aminotransferase; TBIL, total bilirubin; ALB, albumin; GGT, γ-glutamyltransferase; ALP, alkaline phosphatase; CRP, C-reactive protein; WBC, white blood cell; NE, neutrophilicgranulocyte count; LE, lymphocyte count; NLR, neutrophil to lymphocyte ratio; PLT, platelet; PT, prothrombin time; PTA, prothrombin time activity; INR, International Normalized Ratio; DD, D-dimer; AFP, alpha-fetoprotein; PVTT, portal vein tumor thrombus; ICVTT, inferior vena cava tumor thrombus; HVTT, hepatic vein tumor thrombus; CR, Complete Remission.

### 3.2 Analysis of risk factors for prognosis in patients with HCC

To identify the prognostic factors affecting the efficacy of intermediate and advanced HCC patients, we performed univariate and multivariate Cox regression analysis in the training cohort (**Table 2**). Univariate analysis showed that TBIL, ALB, PT, PTA, INR, DD, CD4+ T cell count, PVTT, tumor number≥3, and lymph node invasion were significant prognostic factors. After removing the confounding factors, the results of multivariate analysis showed that TBIL (aHR = 1.02, 95% CI: 1.01-1.03, *P* < 0.001), DD (aHR = 1.07, 95% CI: 1.03-1.12, *P* = 0.001), and PVTT (aHR = 1.61, 95% CI: 1.07-2.43, *P* = 0.022) were independent poor prognostic factors. Based on these results, the linear predictor of the Cox model was constructed as: 0.02 × TBIL + 0.07 × DD + 0.48 × PVTT (with PVTT coded as 0 for absence and 1 for presence). Other clinical variables such as gender, age, transaminase level, tumor diameter, and cirrhosis did not show significant prognostic value (P > 0.05).

**Table 2:** Univariate and multivariate Cox regression analyses in the training cohort.

Variables	Univariate analysis					Multivariate analysis				
	β	S.E	Z	*P*	HR (95%CI)	β	S.E	Z	*P*	aHR (95%CI)
Sex, Female	-0.03	0.23	-0.13	0.897	0.97 (0.62-1.51)					
Age	-0.00	0.01	-0.31	0.759	1.00 (0.98-1.01)					
AST	-0.00	0.00	-1.13	0.259	1.00 (1.00-1.00)					
ALT	-0.00	0.00	-0.19	0.852	1.00 (1.00-1.00)					
TBIL	0.02	0.00	4.14	**<0.001**	1.02 (1.01-1.03)	0.02	0.00	4.25	**<0.001**	1.02 (1.01-1.03)
ALB	-0.05	0.02	-2.70	**0.007**	0.95 (0.92-0.99)	-0.03	0.02	-1.31	0.189	0.97 (0.93-1.02)
GGT	0.00	0.00	0.82	0.414	1.00 (1.00-1.00)					
ALP	0.00	0.00	0.34	0.737	1.00 (1.00-1.00)					
CRP	0.00	0.00	0.10	0.923	1.00 (0.99-1.01)					
WBC	-0.06	0.05	-1.34	0.182	0.94 (0.86-1.03)					
NE	-0.05	0.06	-0.85	0.394	0.95 (0.85-1.07)					
LE	-0.31	0.18	-1.68	0.093	0.73 (0.51-1.05)					
NLR	-0.00	0.04	-0.06	0.955	1.00 (0.91-1.09)					
PLT	-0.00	0.00	-0.67	0.502	1.00 (1.00-1.00)					
PT	0.11	0.05	2.20	**0.028**	1.12 (1.01-1.24)	-1.32	0.89	-1.49	0.137	0.27 (0.05-1.52)
PTA	-0.02	0.01	-2.04	**0.042**	0.98 (0.97-0.99)	0.01	0.03	0.55	0.586	1.02 (0.96-1.07)
INR	1.23	0.55	2.23	**0.026**	3.42 (1.16-10.09)	15.08	10.21	1.48	0.140	3534966.07 (0.01- 1740023596361873.50)
DD	0.06	0.02	3.35	**<0.001**	1.07 (1.03-1.11)	0.07	0.02	3.20	**0.001**	1.07 (1.03-1.12)
AFP	-0.00	0.00	-0.92	0.358	1.00 (1.00-1.00)					
CD8+ T cell	-0.00	0.00	-1.14	0.256	1.00 (1.00-1.00)					
CD4+ T cell	-0.01	0.00	-2.52	**0.012**	0.99 (0.99-0.99)	-0.00	0.00	-1.83	0.067	1.00 (1.00-1.00)
CR	-0.00	0.01	-0.31	0.755	1.00 (0.99-1.01)					
PVTT										
NO					1.00 (Reference)					1.00 (Reference)
YES	0.64	0.20	3.24	**0.001**	1.89 (1.29-2.78)	0.48	0.21	2.30	**0.022**	1.61 (1.07-2.43)
HVTT										
NO					1.00 (Reference)					
YES	-0.12	0.28	-0.41	0.680	0.89 (0.52-1.54)					
ICVTT										
NO					1.00 (Reference)					
YES	0.05	0.37	0.13	0.895	1.05 (0.51-2.17)					
Tumor number										
< 3					1.00 (Reference)					1.00 (Reference)
≥ 3	0.64	0.20	3.22	**0.001**	1.91 (1.29-2.82)	0.39	0.22	1.76	0.079	1.47 (0.96-2.27)
Tumor diameter, cm										
< 5					1.00 (Reference)					
≥ 5	0.20	0.25	0.82	0.413	1.23 (0.75-2.00)					
Lymph node invasion										
NO					1.00 (Reference)					1.00 (Reference)
YES	0.49	0.21	2.33	**0.020**	1.63 (1.08-2.46)	0.39	0.23	1.71	0.087	1.48 (0.95-2.32)
Liver cirrhosis										
NO					1.00 (Reference)					
YES	0.31	0.27	1.13	0.259	1.36 (0.80-2.32)					

HR, hazard ratio; aHR, adjusted hazard ratio; CI, confidence interval; ALT, alanine aminotransferase; AST, aspartate aminotransferase; TBIL, total bilirubin; ALB, albumin; GGT, γ-glutamyltransferase; ALP, alkaline phosphatase; CRP, C-reactive protein; WBC, white blood cell; NE, neutrophilicgranulocyte count; LE, lymphocyte count; NLR, neutrophil to lymphocyte ratio; PLT, blood platelet; PT, prothrombin time; PTA, prothrombin time activity; INR, International Normalized Ratio; DD, D-dimer; AFP, alpha-fetoprotein; CR, Complete Remission; PVTT, portal vein tumor thrombus; ICVTT, inferior vena cava tumor thrombus.

Bold values indicate statistical significance (P < 0.05).

**Figure 2:**
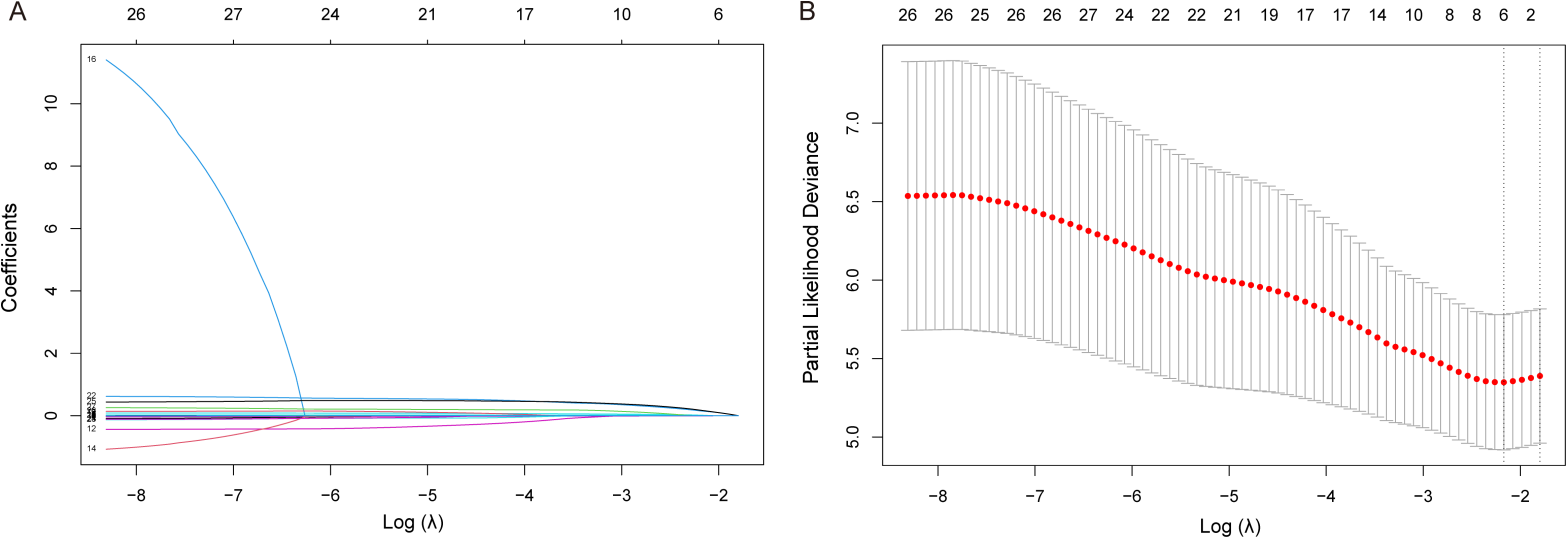
LASSO regression analysis for prognostic factor selection. **(A)** LASSO coefficient profiles of the candidate variables. **(B)** Cross-validation for tuning parameter selection in the LASSO model.

In addition, we performed Lasso regression analysis in the training cohort (**Figure 2**). **Figure 2A** shows the changing trajectories of the coefficients of different independent variables as the log(λ) value changes. When log(λ) increases, the coefficients of most variables gradually tend to zero, indicating that these variables are excluded by the model. **Figure 2B** shows the change of partial likelihood deviation with log(λ) during cross-validation, where the vertical dashed line indicates the position of the optimal λ value. At the optimal λ value, a total of 10 variables were retained by the Lasso model. AST, TBIL, ALB, LE, DD, AFP, CD4+ T cell count, PVTT, tumor number (≥3), and lymph node invasion were identified as key factors affecting patient prognosis.

The time-dependent AUC values for the Cox regression model showed considerable variability across different time points and cohorts. In the training cohort, the AUCs were 0.69, 0.65, and 0.71 at 6, 12, and 24 months, respectively. In the validation cohort, the corresponding AUCs were 0.73, 0.71, and 0.45, with a marked drop at 24 months (**Table 3**). In contrast, the LASSO-derived model demonstrated stable performance, with C-index values remaining consistent across all time points in both cohorts. Specifically, the C-index values for the LASSO model were 0.74, 0.72, and 0.72 at 6, 12, and 24 months in the training cohort, and 0.73, 0.72, and 0.72 in the validation cohort (**Table 4**). These results indicate that the LASSO-derived model exhibited superior stability and generalizability compared to the traditional Cox regression model. A nomogram was subsequently constructed based on the 10 variables selected by LASSO regression. Although baseline differences existed between the training and validation cohorts, it is important to note that the variables showing imbalance (lymphocyte count, NLR, PT, PTA, INR) were not selected as predictors in the final prognostic nomogram.

**Table 3:** The time-dependent ROC curves of the Cox nomogram predicting overall survival.

Time Point	Total	Training set	Validation set
6 Months	0.69	0.73	0.56
12 Months	0.65	0.71	0.62
24 Months	0.71	0.60	0.45

**Table 4:** The C-index of the LASSO nomogram predicting overall survival.

Time Point	Training cohort	Validation cohort
6 Months	0.74	0.73
12 Months	0.72	0.72
24 Months	0.72	0.72

### 3.3 Establishment and validation of the prognostic nomogram for HCC patients

**Figure 3:**
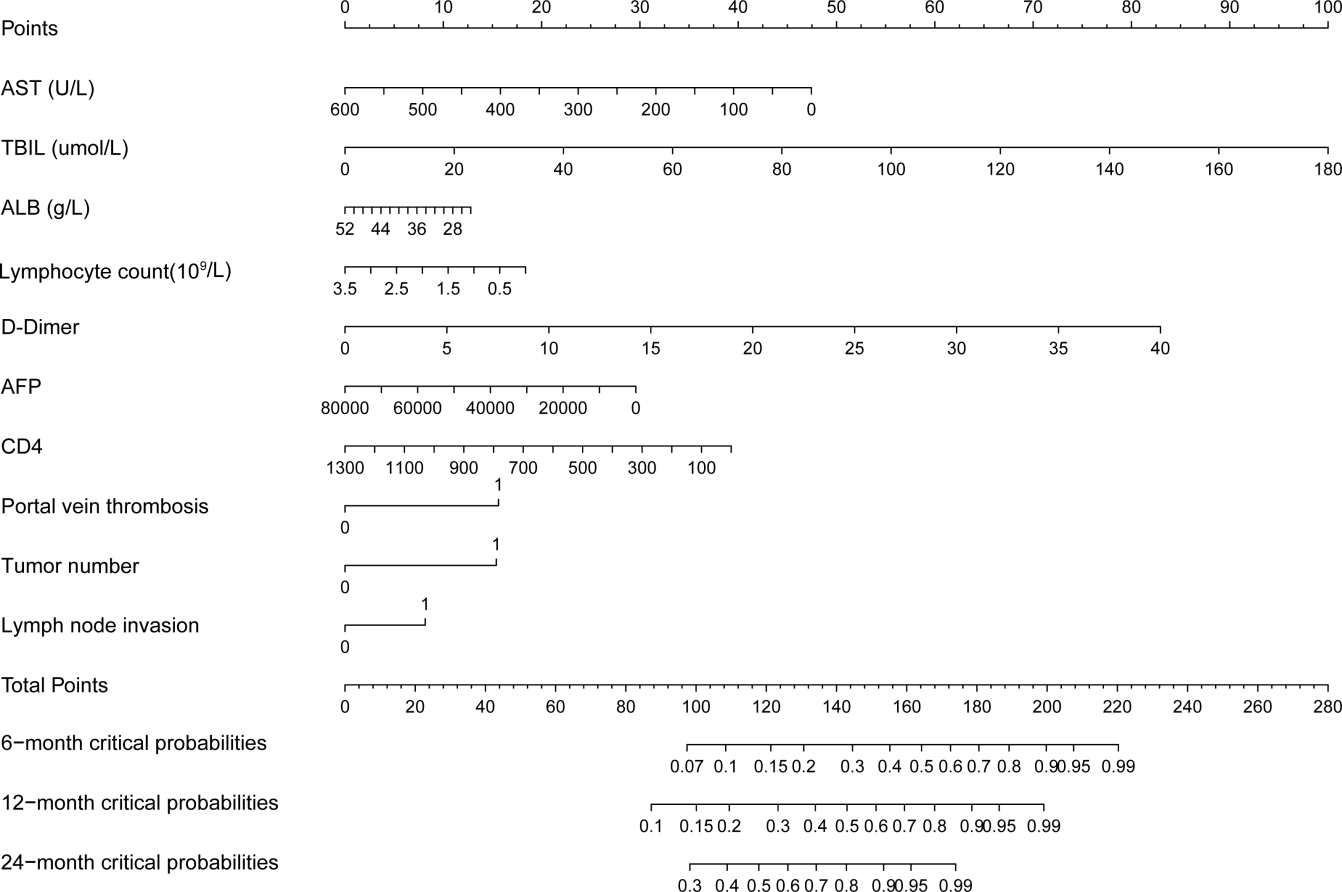
Nomogram for predicting tumor progression in HCC patients. Nomogram integrating 10 prognostic factors to predict 6-month, 12-month, and 24-month tumor progression probability.

**Figure 4:**
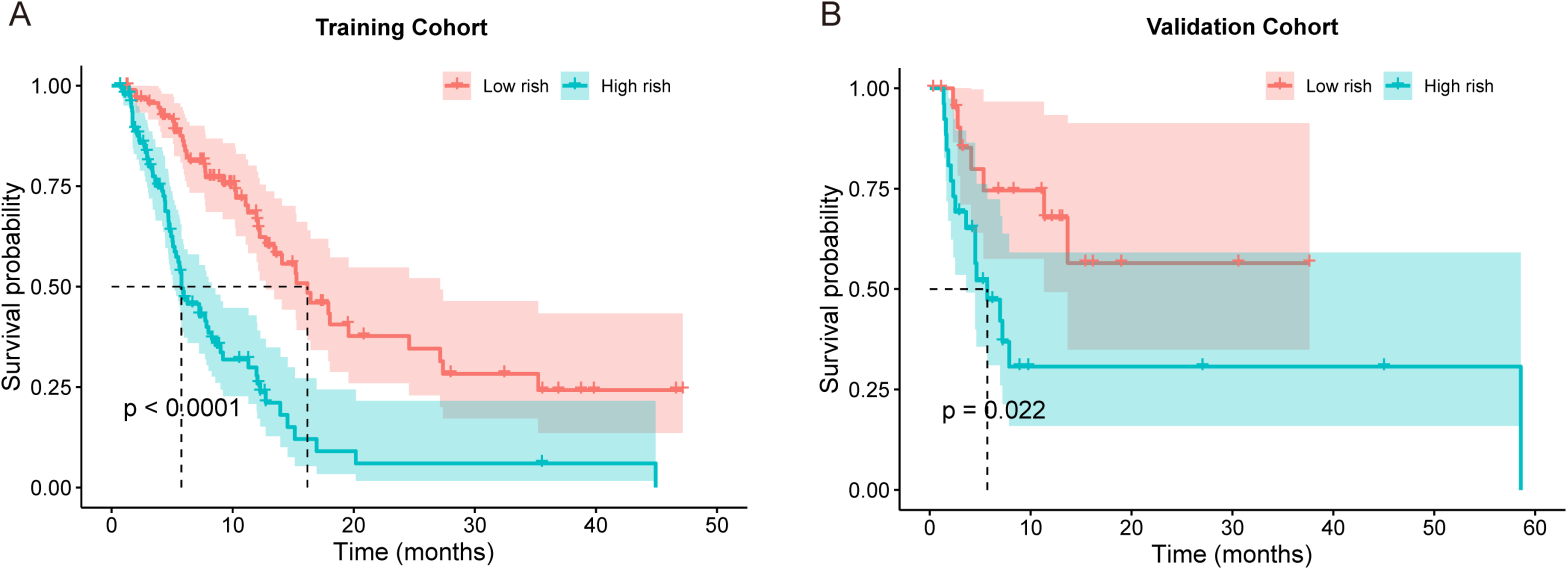
Kaplan-Meier survival curves stratified by risk groups. The survival rates between low-risk and high-risk groups in training cohort **(A)** and validation cohort **(B)**.

Based on the clinical risk factors screened by LASSO regression analysis, we constructed a nomogram model to predict the tumor progression of intermediate and advanced HCC patients (**Figure 3**). The model integrated parameters, such as AST, TBIL, ALB, lymphocyte count, DD, AFP, CD4+ T cell count, PVTT, tumor number and lymph node invasion, and predicted the tumor progression probability of patients at 6-, 12- and 24-months. The nomogram demonstrated moderate discriminative ability in the training cohort with C-index values remained relatively consistent at 0.72-0.73 across all time points. According to the total points calculated by the nomogram, patients were divided into low- and high-risk group. Kaplan-Meier survival curve analysis showed that the survival rate of the low-risk group in both the training cohort (**Figure 4A**) and the validation cohort (**Figure 4B**) was significantly higher than those of the high-risk group (all Log rank *P* < 0.05). Specifically, patients with a total nomogram score of less than 145 were categorized as the low−risk group, while those with a score of 145 or higher were categorized as the high−risk group.

### 3.4 Model prediction performance evaluation

**Figure 5:**
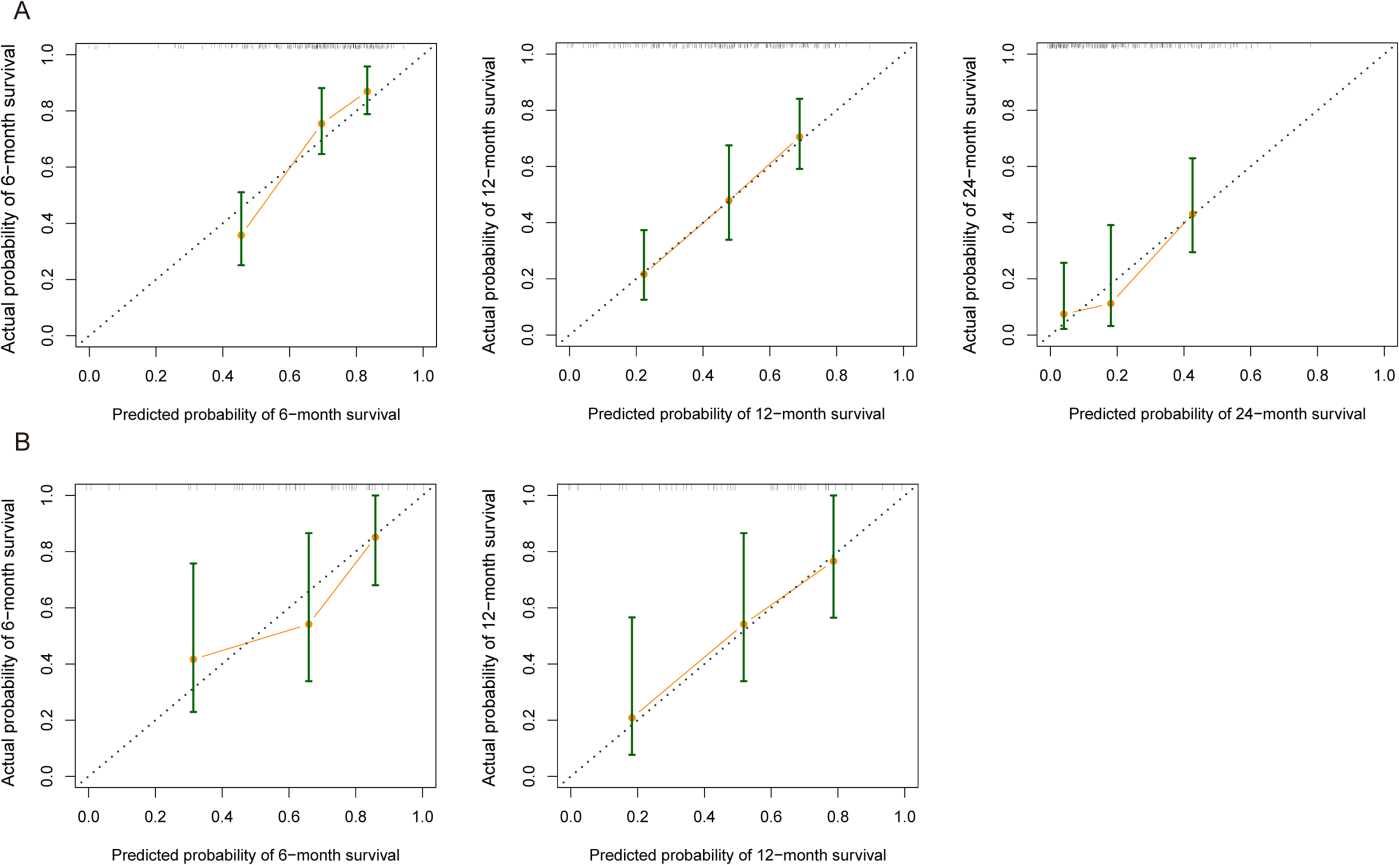
Calibration curves for nomogram performance evaluation. Calibration curves of 6-month, 12-month, and 24-month tumor progression predictions in training cohort **(A)** and validation cohort **(B)**.

**Figure 6:**
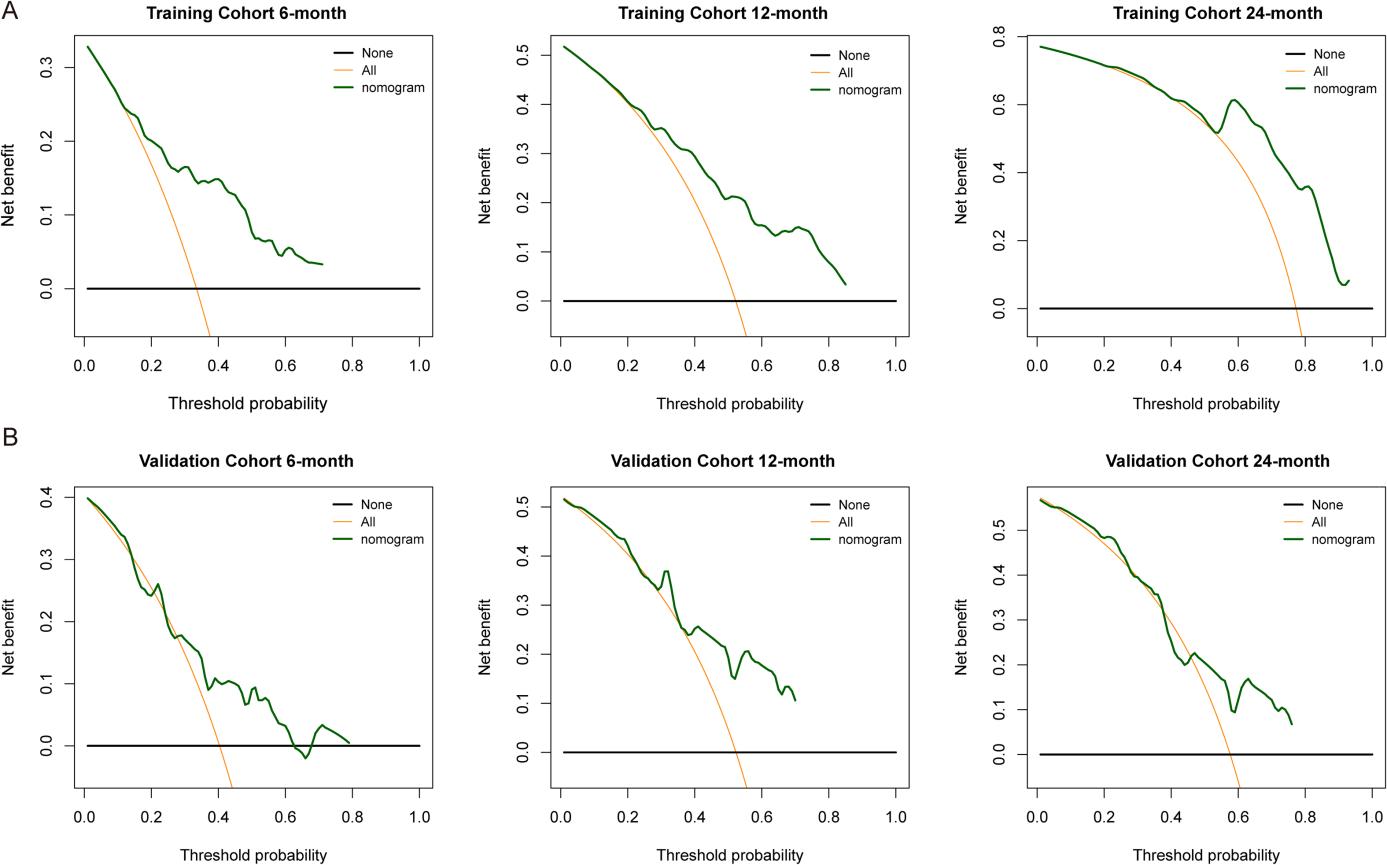
Decision curve analysis for clinical utility assessment. Decision curve analysis of 6-month, 12-month, and 24-month tumor progression predictions in training cohort **(A)** and validation cohort **(B)**.

We evaluated the prediction accuracy of the prediction model in the training cohort and validation cohort. In the training cohort (**Figure 5A**), the 12-month probability predicted by the model was highly consistent with the actual observed probability, and the actual probability of each prediction probability group were basically distributed near the ideal calibration line. The prediction of 6-month and 24-month probabilities also showed good calibration performance. The robustness of the model was further supported in the validation cohort (**Figure 5B**). We further performed DCA in the training and validation cohort. **Figure 6A** shows the net benefit curve of the training cohort in 6-, 12- and 24-month prediction. In most threshold probability ranges (0.2-0.5), our prediction model provides higher net benefits than the “all treatment” or “all no treatment” strategy. In the validation cohort (**Figure 6B**), the model also showed considerable clinical decision-making value.

### 3.5 Nonlinear relationship analysis

**Figure 7:**
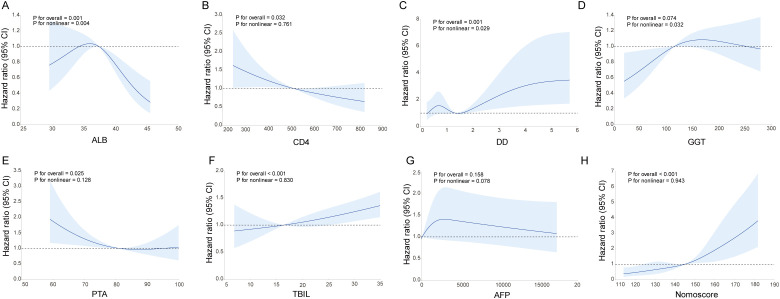
Restricted cubic spline analysis of nonlinear relationships.Nonlinear risk relationship curves between ALB **(A)**, CD4+ T cell count **(B)**, DD **(C)**, GGT **(D)**, nomogram score **(E)**, PTA **(F)**, AFP **(G)**, TBIL **(H)** and tumor progression. TBIL, total bilirubin; ALB, albumin; GGT, γ-glutamyltransferase; PTA, prothrombin time activity; DD, D-dimer; AFP, alpha-fetoprotein.

Restricted cubic spline analysis was used to evaluate the relationship between multiple clinical biochemical indicators and the tumor progression of intermediate and advanced HCC patients. **Figure 7** showed the nonlinear risk relationship curves of each parameter. ALB level was significantly nonlinearly correlated with patient prognosis (*P* overall = 0.001, *P* nonlinear = 0.004). When the ALB level was in the range of about 33–37 g/L, the hazard ratio reached a peak; when ALB was lower than 33 g/L or higher than 37 g/L, the hazard ratio decreased significantly (**Figure 7A**). DD showed a significant nonlinear correlation with prognosis (*P* overall = 0.001, *P* nonlinear = 0.029). In particular, when DD was greater than 2, the hazard ratio increased sharply (**Figure 7C**). Although GGT did not reach a strictly statistically significant level in the overall correlation (*P* overall = 0.074), its nonlinear relationship was significant (P nonlinear = 0.032) (**Figure 7D**). There was no significant nonlinear relationship between CD4+ T cell count, nomoscore, PTA, AFP, and TBIL and patient disease progression (all *P* nonlinearity ≥ 0.05) (**Figure 7B, E–H**).

## 4 Discussion

In this study, a clinical prediction model for the treatment effect of TACE combined with lenvatinib and sintilimab was constructed based on a cohort of Chinese intermediate and advanced HCC patients. By integrating clinical biochemical indicators and tumor characteristics, we established a nomogram model with good predictive performance, which can effectively distinguish the population that will benefit from treatment.

Although targeted immunotherapy has greatly improved patient survival, some patients still cannot benefit from treatment, and the incidence of adverse reactions cannot be ignored. Therefore, developing an effective prediction model that can screen the best beneficiary population is of urgent significance for clinical practice. Our study showed that AST, TBIL, ALB, lymphocyte count, DD, AFP, CD4+ T cell count, PVTT, tumor number (≥3), and lymph node invasion were independent adverse prognostic factors in intermediate and advanced HCC patients. The nomogram model constructed based on these factors showed moderate predictive performance in both training cohort and validation cohort. Decision curve analysis suggested that within certain threshold probability ranges (0.2-0.5), the model provided a net benefit exceeding that of the “treat all” or “treat none” strategies, indicating potential clinical utility. While the model may offer some value for risk stratification, its limited robustness currently precludes direct clinical application without further external validation.

Inflammation and coagulation function are closely related to the occurrence and development of HCC and the efficacy of immunotherapy (25–27). High levels of TBIL reflect the degree of liver function impairment, which may affect drug metabolism and immune system function. Pan S et al. found that hyperbilirubinemia were associated with reduced efficacy of PD-1 inhibitors, which is consistent with our results (28). DD, as a marker of the body’s fibrinolytic function, showed a significant nonlinear relationship with patient prognosis in our study, especially when DD was greater than 2, the hazard ratio increased sharply. Abnormal coagulation function may promote tumor progression and affect the effect of immunotherapy (29). The presence of PVTT not only indicates that the tumor has aggressive biological behavior, but may also lead to portal hypertension and liver function deterioration, thereby affecting the treatment effect (30, 31). A multicenter retrospective-prospective study found that PVTT is also an independent predictor of variceal bleeding and death in patients with hepatocellular carcinoma (32).

The differential expression of CD8+ T cells, CD4+ T cells, and exhausted T cells is a key target for improving the efficacy of immunotherapy for HCC (33). Dynamic changes in peripheral blood CD8+ T cells are prognostic biomarkers in HCC patients treated with atezolizumab combined with bevacizumab (34). As a key component of the immune microenvironment, the number and functional status of CD4+ or CD8+ T cells may affect the efficacy of PD-1 inhibitors. Our model incorporates CD4+ T cell count as a predictive factor for HCC patients. Zuo A, et al. also found that the ratio of resident to exhausted CD4+ T cells is expected to be a potential biomarker for HCC prognosis and immunotherapy response (35). However, it is important to acknowledge that peripheral blood CD4+ T cell counts serve only as an indirect surrogate of intratumoral immune dynamics. The liver functions not only as a metabolic organ but also as a central immunological site characterized by a delicate balance between immune tolerance and immune activation. Hepatocytes constitutively express major histocompatibility complex (MHC) class I molecules, enabling presentation of endogenous peptides derived from viral proteins and tumor-associated antigens to CD8+ cytotoxic T lymphocytes. In chronic hepatitis B infection and hepatocellular carcinoma, alterations in the antigen processing machinery—including proteasomal degradation and peptide transport—may reduce effective MHC class I presentation and facilitate immune escape (36). Parallel to this, antigen-presenting cells resident in the liver, including Kupffer cells and liver sinusoidal endothelial cells, can express MHC class II molecules under inflammatory conditions, presenting antigens to CD4+ helper T cells (37). The equilibrium between MHC class I-mediated cytotoxic responses and MHC class II-mediated helper or regulatory responses critically influences the efficacy of immune checkpoint inhibitors. Autoimmune hepatitis provides a contrasting paradigm in which antigen presentation becomes excessively immunogenic. In that setting, both MHC class I- and class II-mediated presentation drives destructive T cell responses against hepatocytes, highlighting how dysregulation of antigen processing and presentation can shift the liver from a tolerogenic to an inflammatory state. In hepatocellular carcinoma, the tumor often exploits the tolerogenic nature of the liver, downregulating MHC class I expression or inducing local immunosuppression (38). Locoregional therapies such as TACE may induce tumor necrosis and increase antigen release, potentially enhancing antigen presentation and modulating immune recognition—a consideration relevant to our study population. Therefore, peripheral blood CD4+ T cell counts, while pragmatically valuable as readily accessible clinical markers, should be interpreted within this broader framework of hepatic antigen processing and presentation. They serve as indirect surrogates rather than direct reflections of the complex intrahepatic immune dynamics. Future prognostic models may benefit from incorporating indicators of antigen presentation efficiency or immune activation status, rather than relying solely on peripheral cell counts.

Although the prediction model constructed in this study showed good predictive ability based on existing clinical indicators, with the development of genomics and multi-omics technologies, the model is expected to further improve the prediction accuracy by integrating more molecular markers in the future. High-throughput sequencing technology can identify gene mutations and expression profiles associated with the response to targeted immunotherapy (39–41). Wong CN et al. used targeted next-generation sequencing technology and found that lower tumor mutation burden (TMB) may limit the role of TMB as a predictor of HCC immunotherapy efficacy (42). Another study highlighted the key role of lipid metabolism in HCC progression by single-cell RNA sequencing and WGCNA and identified PTGES3 as a potential prognostic biomarker and therapeutic target (43). In addition, biomarkers related to tumor microenvironment characteristics and immune function may also provide incremental predictive value for the model. Wu J et al. constructed a risk model of NK cell-related genes through weighted gene co-expression network analysis (WGCNA) and single-cell sequencing RNA to predict the immunotherapy response of HCC patients (41). Cao L et al. constructed a 12-gene signature based on CD8+ T cell heterogeneity to predict survival outcome and immunotherapy response in hepatocellular carcinoma (44). Another single-cell analysis showed that tumor-associated neutrophils can affect the function of macrophages, NK cells, and T cells, and the model of tumor-associated neutrophil-related genes can also be used to predict patient prognosis and immunotherapy response (45).

The restricted cubic spline analysis revealed intriguing nonlinear relationships between albumin (ALB), D-dimer (DD), and progression risk, which warrant further mechanistic interpretation. The “U-shaped” association for ALB, with a risk peak within the low-normal range (33–37 g/L), may extend beyond mere nutritional status. It might reflect a specific systemic inflammatory or metabolic state under treatment pressure, where a slight decline from standard levels is associated with activated pathways of tumor progression and therapy resistance. Conversely, very low ALB indicates poor hepatic synthetic function, while higher levels may denote better overall physiological reserve. Regarding DD, the sharp increase in hazard ratio beyond the threshold of 2 µg/mL is particularly notable. This likely signifies a transition into a state of overt hypercoagulability, which is increasingly recognized not merely as a bystander effect but as an active contributor to tumor progression, metastasis, and immunotherapy resistance through mechanisms such as facilitating tumor cell survival, shielding from immune surveillance, and promoting an immunosuppressive microenvironment. These nonlinear patterns suggest the existence of distinct pathophysiological sub-phenotypes within the intermediate/advanced HCC population undergoing combination therapy. Identifying these thresholds (e.g., ALB ~35 g/L, DD = 2 µg/mL) could help clinicians stratify patients not only by risk but also by potential underlying biology, possibly guiding adjunctive interventions such as targeted nutritional support or evaluation for antithrombotic therapy in future clinical investigations. Nevertheless, it is important to acknowledge that these spline-based findings were derived from a modest sample size (n=194 in the training cohort). Complex nonlinear relationships identified through flexible modeling techniques such as restricted cubic splines can be sensitive to sample size and may be prone to overfitting or chance findings. Therefore, while these observations provide intriguing biological insights, they should be considered hypothesis-generating rather than definitive. External validation in larger, independent cohorts is essential to confirm the robustness and generalizability of these nonlinear associations before they can be reliably integrated into clinical decision-making.

This study has some limitations. First, the retrospective, single-center design and reliance on internal validation fundamentally limit generalizability. Patient selection may have been influenced by institutional protocols and physician discretion, potentially introducing selection bias. Consequently, our cohort may not fully represent the broader, heterogeneous population of intermediate-to-advanced HCC patients encountered in diverse clinical practices. The current validation strategy—an internal 8:2 split yielding a small validation cohort (n=49) with baseline imbalances—is insufficient to establish model robustness, as such internal validation can overestimate performance. Although this ratio is commonly employed in prognostic model development to prioritize sufficient statistical power for robust model training while reserving a portion of data for initial internal validation. Secondly, it is important to consider the methodological aspect of variable selection. The use of LASSO regression, while effective for deriving a parsimonious model from correlated predictors, may exhibit some instability in the specific set of selected features when applied to a cohort of our size. This inherent sensitivity to sample composition could affect the interpretative consistency of the model. Future work employing repeated resampling techniques (e.g., bootstrapped LASSO) or comparing feature importance with ensemble methods (e.g., Random Forest) on larger datasets would be valuable to verify the robustness of the identified prognostic variables. Thirdly, the prediction model was constructed using baseline clinical and laboratory parameters, prioritizing factors readily available in routine clinical practice to facilitate initial application. While this pragmatic approach enhances immediate usability, it does not capture the full biological complexity of HCC, including tumor molecular heterogeneity, the dynamic immune microenvironment, or detailed radiomic phenotypes. Future iterations of this model could be significantly enhanced by integrating multi-dimensional data layers, such as radiomic features from medical imaging, circulating tumor biomarkers (e.g., cell-free DNA, microRNAs), and genomic characteristics (e.g., tumor mutational burden). The application of machine learning techniques capable of handling such high-dimensional data would be a logical next step to develop a more comprehensive and biologically anchored prognostic tool. Fourthly, the follow-up time was limited, and the predictive value of the model for long-term survival could not be evaluated. Fifthly, it should also be noted that our model predicts the time to radiological progression. While this is a direct and clinically actionable measure of treatment failure, it may not fully capture patient benefit in the era of immunotherapy, where atypical response patterns such as pseudoprogression—an initial increase in tumor burden followed by later response—can occur. Radiologic progression alone might therefore overestimate treatment failure in some patients. Incorporating composite endpoints such as progression-free survival (encompassing both radiological progression and clinical deterioration) or overall survival in future validation studies would provide a more comprehensive assessment of the model’s utility in guiding overall patient management. Sixth, due to the retrospective nature of this study, several potentially important confounders could not be assessed. These include dynamic changes in viral load (particularly relevant for patients with HBV-related HCC), the occurrence and severity of specific immune-related adverse events (which may reflect immune activation and correlate with treatment response), and treatment adherence or dose modifications. The absence of these variables may introduce residual confounding and limit the interpretability of our findings.

Future research should prioritize rigorous external validation of this model, including temporal validation using patients from different time periods to account for shifts in clinical practice, and geographic validation utilizing independent multicenter cohorts with varying demographics and disease characteristics. Only through such diverse assessments can the true transportability of this prediction model be confirmed. To ensure reliable endpoint evaluation in the immunotherapy era, future validation studies should ideally incorporate central radiologic review to differentiate true progression from pseudoprogression. Additionally, while no established benchmark model currently exists for direct comparison in this specific patient population (undergoing TACE combined with lenvatinib and sintilimab), the emergence of new prognostic tools will enable formal assessment of incremental value using metrics such as Net Reclassification Improvement (NRI) and Integrated Discrimination Improvement (IDI). Beyond validation, the ultimate goal is to transcend the limitations of conventional indicators by constructing a truly multidimensional model that captures HCC’s biological complexity. This will require systematic integration of radiomic features from medical imaging, dynamic circulating biomarkers (e.g., cell-free DNA, microRNAs, and longitudinal AFP/DCP profiles), and molecular profiling data (e.g., tumor mutational burden, immune gene signatures). Leveraging machine learning to synthesize these high-dimensional, multi-modal data layers will be essential for developing a more biologically grounded, accurate, and clinically actionable prediction tool.

## 5 Conclusions

This study constructed a clinical prediction model that can predict the treatment effect of TACE combined with lenvatinib and sintilimab in patients with intermediate and advanced HCC. The model integrates multidimensional indicators such as liver function, coagulation function, tumor characteristics and immune function, showing promising predictive performance and clinical application value. It helps to screen the best beneficiaries, optimize treatment strategies and improve resource utilization efficiency.

## Data Availability

The original contributions presented in the study are included in the article/supplementary material. Further inquiries can be directed to the corresponding authors.
